# Acute graft versus host disease 1976–2020: reduced incidence and predictive factors

**DOI:** 10.3389/fmed.2023.1320692

**Published:** 2024-01-24

**Authors:** Alessandra Di Francesco, Anna Maria Raiola, Alida Dominietto, Carmen Di Grazia, Francesca Gualandi, Maria Teresa Van Lint, Stefania Bregante, Patrizia Chiusolo, Luca Laurenti, Federica Sora, Sabrina Giammarco, Elisabetta Metafuni, Alberto Fresa, Simona Sica, Emanuele Angelucci, Andrea Bacigalupo

**Affiliations:** ^1^Dipartimento di Diagnostica per Immagini, Radioterapia Oncologica ed Ematologia, Fondazione Policlinico Universitario A. Gemelli IRCCS, Roma, Italy; ^2^IRCCS AOU San Martino IST, Genova, Italy; ^3^Sezione di Ematologia, Dipartimento di Scienze Radiologiche ed Ematologiche, Università Cattolica del Sacro Cuore, Roma, Italy

**Keywords:** allotransplantation, GvHD, acute GVHD, transplant, hematology

## Abstract

We studied the incidence of acute graft versus host disease (GvHD) and its outcome in three consecutive time frames (year <2000; 2000–2010; >2010), in 3,120 patients allografted in two transplant Centers between 1976 and 2020. The median age increased over the three periods from 32 to 42 to 54 years (*p* < 0.00001). The median day of onset of GvHD in the three periods was day +14, day +16, and day +30, respectively (*p* < 0.0001). The cumulative incidence (CI) of GvHD grades II–IV in the three periods was 47, 24, and 16%, respectively (*p* < 0.00001). The CI of GvHD grades III–IV was 13, 5, and 4% (*p* < 0.001). In multivariate analysis, significant predictive factors for GvHD II–IV, on top of year of transplant, were anti-thymocyte globulin (ATG) (RR 0.67, *p* > 0.001); post-transplant cyclophosphamide (PTCY) (RR 0.41, *p* < 0.001), a family mismatched donor (RR 1.31, *p* = 0.03) a matched unrelated donor (RR 2.1, *p* < 0.001), an unrelated mismatched donor (RR1.8, *p* = 0.001), donor age above 40 years (RR 1.27, *p* < 0.001), hematological malignancy—as compared to aplastic anemia (RR 2.3, *p* < 0.001). When selecting only GvHD grade II, in a multivariate analysis, there was a significant reduction of transplant-related mortality (TRM) for patients grafted in 2001–2010 (RR 0.62, *p* < 0.0001) and for patients grafted in 2011–2020 (RR 0.35, *p* < 0.0001) as compared to grafts before the year 2000. A similar reduction in time was seen for patients with GvHD grades III–IV. The overall TRM in the three periods was 30, 22, and 16% (*p* < 0.0001) and survival was 47, 51, and 58% (*p* < 0.0001). Relapse risk was unchanged. In conclusion, we showed improved prevention of acute GvHD with time, together with a significant delay in the onset of the disease. Treatment of GvHD has also improved over time, as suggested by both reduced TRM and improved survival in more recent transplant periods.

## 1 Introduction

Acute graft versus host disease (GvHD) is a major complication of allogeneic HSCT and represents a significant cause of morbidity and mortality (1). The incidence of acute GvHD grades II–IV varies according to GvHD prophylaxis, and to the propensity to use *in vivo* T cell depletion with anti-thymocyte globulin (ATG) (1, 2). Other risk factors for acute GvHD are HLA mismatch, together with increased age of both donor and recipient, gender disparity, multiparous female donor, the intensity of the conditioning regimen, and the source of the graft (2, 3). GvHD prophylaxis has changed significantly over the past decades (4, 5) and one would expect the incidence and severity of acute GvHD to be reduced in current transplant programs: In one study, the incidence of GVHD grades II–IV and III–IV was 40 and 19% for patients grafted in 1990–1995, versus 28 and 11%, respectively, in those grafted during the period 2011–2015 (2). Acute GvHD used to be defined as an event occurring within day +100 after transplantation: the National Institute of Health Consortium has redefined GvHD as classic, late, and overlap with chronic GvHD, with no time constraint (6). An exception to the latter statement is hyperacute GvHD, which occurs within the first 2 weeks after transplant and has been associated with HLA mismatch, lower response rate to first-line therapy, and higher mortality (7). Acute GVHD is clinically graded as I to ΙV, based on the extent of skin, liver, and gut involvement, according to the well-known Glucksberg criteria (8); a refined grading system has recently been proposed by the Mount Sinai Acute GvHD Consortium (MAGIC) consortium (9). The grade of acute GvHD has a significant impact on transplant-related mortality (TRM) with wide variation from less than 10% for grade I to over 50% for grade IV (1–4). The combination of a calcineurin inhibitor (such as cyclosporin-CSA or tacrolimus-FK) with methotrexate (MTX) is considered standard prophylaxis (10), but the recent introduction of post-transplant high-dose cyclophosphamide (PTCY) (11) is rapidly changing the landscape, as also shown in a recent prospective randomized trial (12).

We are now reporting the cumulative incidence of acute GvHD in three consecutive time periods (<2000; 2001–2010; >2010) in a relatively large cohort of patients, looking at predictive factors and outcomes.

## 2 Methods

This is a retrospective study of patients allografted in two transplant Centers: Ospedale San Martino Genova and Policlinico Gemelli, Rome. Patients gave their informed consent for the collection of transplant data for local, national, and international databases, provided this would be performed for research purposes. Documentation and sharing of transplant procedures are a requisite of National and International Governing Agencies. The data were collected prospectively by physicians in the transplant unit.

The aim of the study was to analyze the cumulative incidence of acute GvHD grades II–IV and grades III–IV in three different time periods: before the year 2000; between 2000 and 2010; and after 2010. In addition, we studied variables that would predict the development and outcome of acute GvHD.

### 2.1 Patients

We analyzed 3,120 patients, 2,510 from Genova San Martino and 610 from Rome Gemelli: 1234 patients were allografted between 1976 and 2000 (39%); 927 from 2000 to 2010 (30%); and 959 after 2010 (31%). The number of patients in the three different time periods, in the Genova and Rome Unit were 113,689; 786,135, and 576,398, respectively. Clinical characteristics of patients, donors, and transplant procedures are outlined in Table 1. In the three time periods, the median age increased from 32 (range 1–66) to 42 years (range 9–71) to 54 years (range 12–74) (<0.00001); the use of family mismatched donors increased from 10 to 54% (*p* < 0.001).

**Table 1:** Patient’s features in the different time frames.

	<2000	2001–2010	>2010	*p*-value
N. patients	1,234	927	959	
Median age	32 (1–66)	42 (9–71)	54 (12–72)	<0.00001
**Diagnosis**				
NM/M diagnosis^a^	104/1,130	46/881	29/930	0.1
Acute leukemia (%)	335 (29)	247 (26)	235 (25)	0.8
Phase-CR (%)	271 (76)	155 (63)	154 (66)	0.0006
CML (%)	400 (32)	72 (7)	18 (2)	<0.0001
MF (%)	8 (0.6)	59 (6)	102 (11)	<0.000001
**GvHD prophylaxis**				
CSA + MTX + ATG (%)^b^	208 (17)	419 (45)	208 (22)	<0.0001
PTCY (%)^c^	0	18 (2)	562 (59)	<00001
CSA + MTX (%)^d^	1,026 (83)	490 (53)	189 (19)	<0.0001
**Donor age**	33 (2–69)	36 (11–75)	35 (13–68)	0.003
**Donor type**				
Sibling (%)	955 (77)	450 (49)	208 (22)	<0.000001
Family MM (%)^e^	123 (10)	95 (10)	520 (54)	<0.000001
Unrelated M (%)^f^	142 (11)	211 (23)	119 (12)	<0.000001
Unrelated MM (%)^g^	15 (3)	171 (18)	112 (12)	<0.000001
**Stem cell source**				
Bone marrow	1,106	593	657	<0.000001
Peripheral blood	128	224	277	<0.000001
Cord blood	0	110	25	<0.000001

NM, non-malignant; M=lalignant:Phase-CR, early phase or complete remission; CML, chronic myeloid leukemia; MF, myelofibrosis; CSA MTX ATG, cyclosporin, methotrexate, anti-thymocyte globulin; family MM, family mismatched; unrelated M, unrelated matched; unrelated MM, unrelated mismatched.

a Non-malignant/malignat diagnosis.

b Ciclosporine + methotrexate + thymoglobuline.

c Post transplantation cyclophosfamide.

d Ciclosporine + methotrexate.

e Family mismatched.

f Unrelated matched.

g Unrelated mismatched.

GvHD prophylaxis has changed over time (Table 1). Before the year 2000, methotrexate- and cyclosporine-based regimens (with or without anti-thymocyte globulin) were the preferred ones. Between 2000 and 2010, 53% of patients underwent prophylaxis with MTX + CsA, 45% with MTX + CsA + ATG, and 2% with post-transplant cyclophosphamide (PTCy).

After the year 2010, the most used regimen was PTCY (PtCy = 59%; MTX + CsA = 20%; MTX + CsA + ATG = 21%) (Table 1).

### 2.2 Statistical analysis

The NCSS 19 for Windows (Kaysville, UT, United States) was used for contingency tables, rank sum test, cumulative incidence (CI) rates, and Kaplan–Meier survival curves. When calculating the CI of transplant-related mortality (TRM), the competing risk was relapsed, and vice versa. When calculating the CI of GvHD, the competing risk was death without GvHD. The log-rank test was used for differences between survival curves; the Grays’ test was used to assess differences between cumulative incidence curves, in univariate analysis. A multivariate Cox analysis on the risk of developing acute GvHD grades II–IV was developed with the following variables: donor and recipient age, conditioning regimen intensity (myeloablative versus reduced intensity), GvHD prophylaxis, donor type, stem cell source, and diagnosis (malignant versus non-malignant). A multivariate Cox model was also used to analyze variables influencing TRM. We also studied the survival and transplant mortality of patients with acute GvHD in the three time periods. The same was performed for the CI of acute GvHD grades III–IV.

## 3 Results

### 3.1 Incidence of acute GvHD

**Figure 1:**
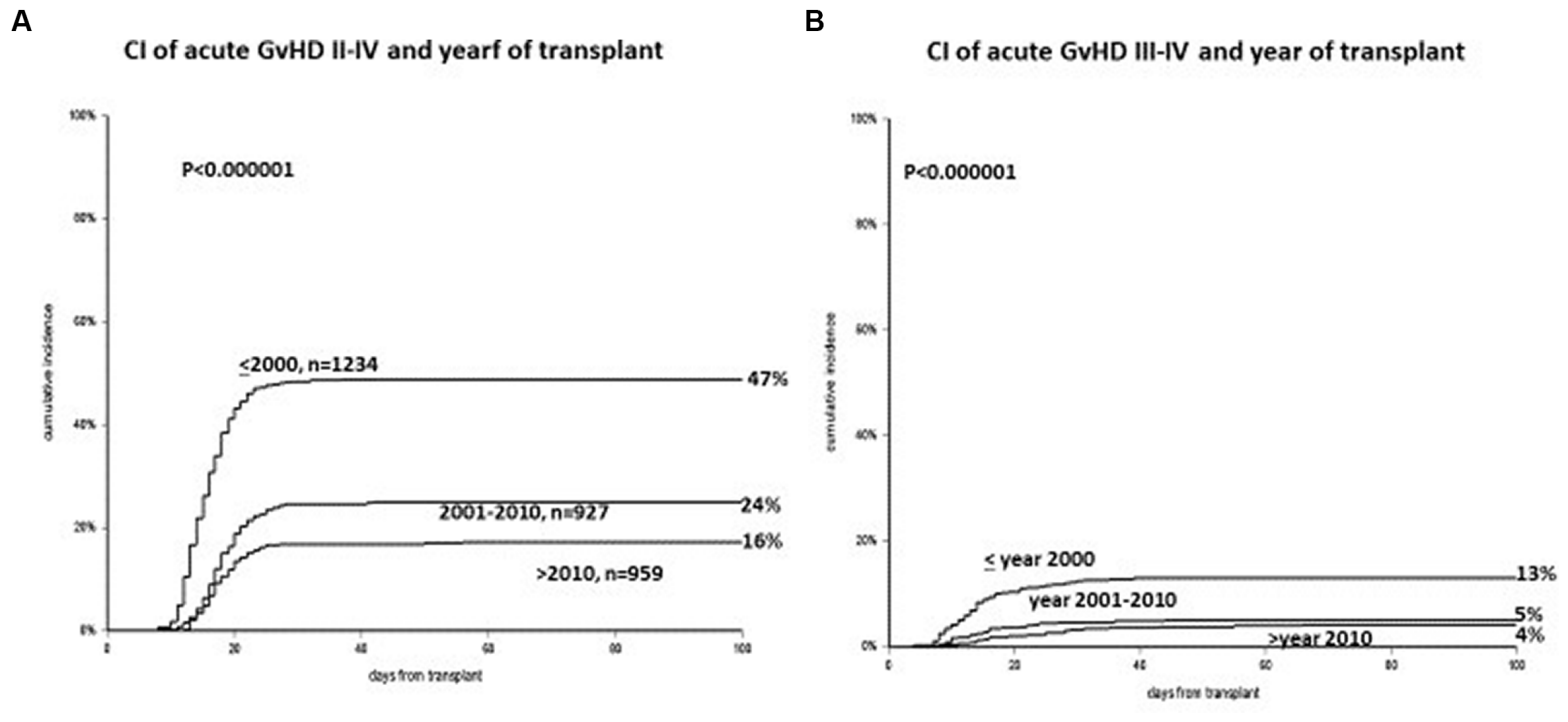
**(A)** Cumulative incidence of acute GvHD grades II–IV, with a significant reduction in three time periods (<2000, 2001–2010, >2010). **(B)** Cumulative incidence of acute GvHD grades III–IV: again a significant reduction in three time periods (<2000, 2001–2010, >2010).

The cumulative incidence (CI) of acute GvHD grades II–IV has changed over the years: it was 47% before 2000, 24% between 2000 and 2010, and 16% after 2010 (*p* < 0.00001) (Figure 1A). The CI of acute GvHD grades III–IV was 13%, 5%, and 4%, respectively (*p* < 0.001) (Figure 1B). The CI of GvHD grade II was 40%, 20%, and 12% during the three periods (*p* < 0.001). The incidence of GvHD grades II–IV and III–IV was almost identical in the two transplant Units, in the three time periods. In a multivariate Cox analysis on the risk of developing GvHD grades II–IV, significant predictors were the year of transplant, the addition of ATG to CSA + MTX for GvHD prophylaxis, the combination PTCY+CSA + MMF, donor type, donor age over 40 years, an unrelated donor as compared to HLA identical siblings, and a malignant disease as compared to aplastic anemia (Table 2). Non-significant predictors were recipients’ age, conditioning intensity (MAC/RIC), donor and recipient gender, and transplant Unit. Comparable data were obtained when looking at the risk of developing grades III–IV acute GvHD. Reduction of GvHD with time was seen in the HLA and also in the HLA mismatched transplants.

**Table 2:** Multivariate logistic regression analysis on GvHD II–IV-significant predictors.

Variable	Baseline	Compared	RR	(95% CI)	*p*-value
Year of TX	<2000	2001–2010	0.37	0.26–0.54	<0.00001
		>2010	0.39	0.24–0.64	0.0002
Prophylaxis	CSA + MTX	+ATG	0.65	0.63–0.79	<0.00001
		PTCY +CSA + MMF	0.42	0.22–0.96	0.03
Donor type	HLA id SIB	Fam.mismatched	1.33	0.83–1.10	0.2
		UD matched	2.1	1.69–2.61	<0.00001
		UD mismatched	1.9	0.92–3.90	0.08
		UD cord blood	0.63	0.14–2.67	0.53
Donor age	<40 years	≥40 years	1.52	1.17–1.99	0.001
Diagnosis	SAA	Malignancies	1.61	1.18–2.20	0.002

Year of Tx, year of transplant; CsA, cyclosporine; MTX, methotrexate; ATG, anti-thymocyte globulin; PTCY, post-transplant cyclophosphamide; UD, unrelated donor; CB, cord blood; SAA, severe aplastic anemia.

### 3.2 GvHD timing

**Figure 2:**
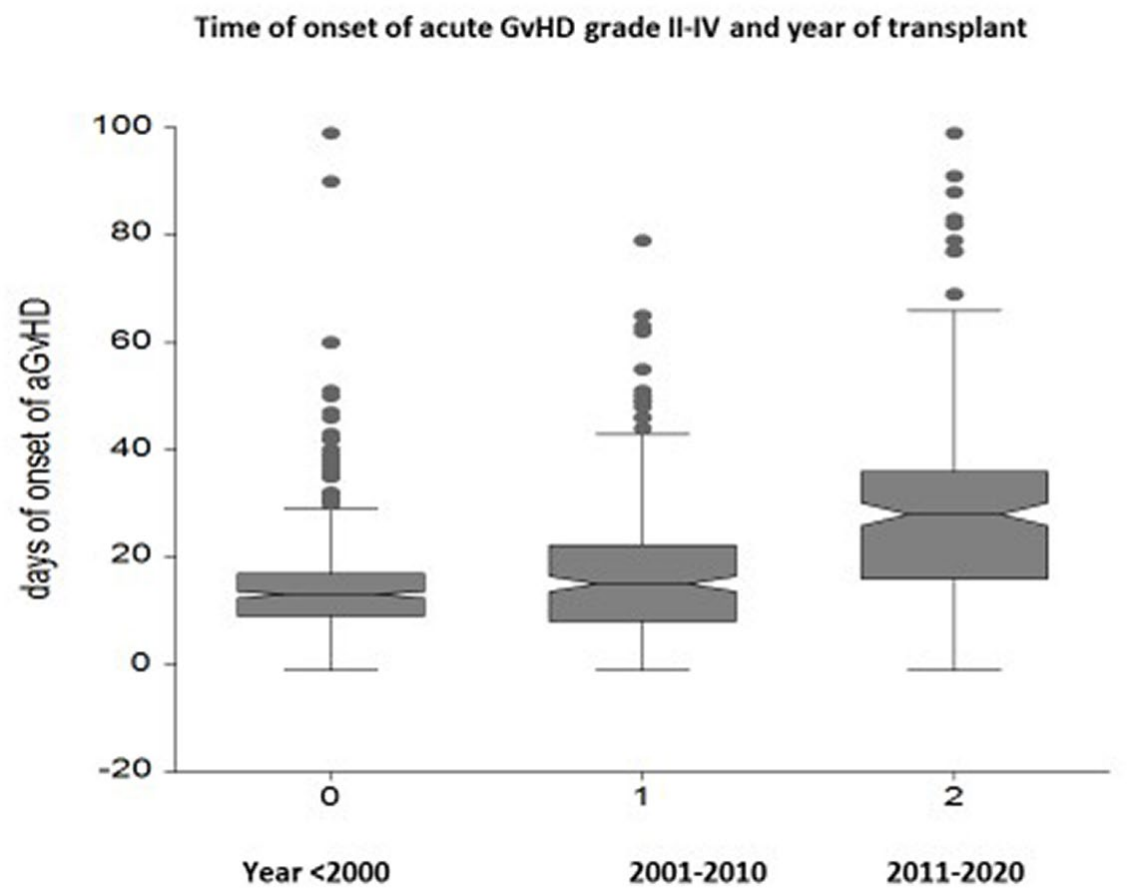
Box plots of the interval between transplantation and the onset of acute GvHD in three different time periods: a delay of GvHD onset is seen from day +14 to day +19 to day +33 in the most recent period (*p* < 0.00001).

The onset of acute GvHD is delayed over time: the median day of onset before the year 2000 was day +14 (range 2–90), between 2001 and 2010 it was day +19 (range 2–98), and beyond the year 2010 it was day +33 (range 2–98) (*p* < 0.0001) (Figure 2). Hyperacute GvHD, developing before day 14 from transplant, was diagnosed in 237 patients before year 2000, 71 patients between 2001 and 2010, and 8 patients beyond 2010 (*p* < 0.0001).

### 3.3 Transplant-related mortality

**Figure 3:**
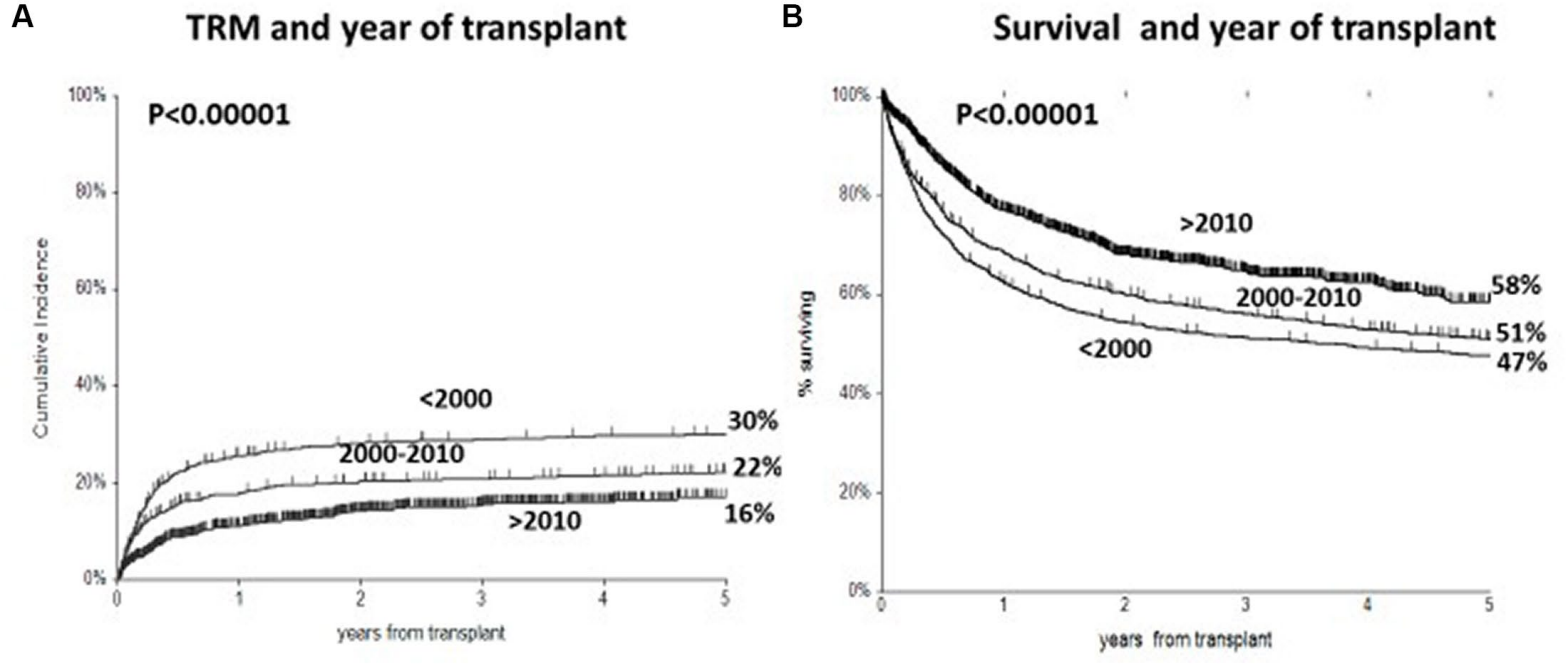
**(A)** Cumulative incidence of transplant-related mortality (TRM) in three time periods, with a significant reduction from 30 to 16% in most recent period. **(B)** Actuarial 5 year survival, with significant improved survival from 47 to 58%.

The 5-year CI of TRM for all patients in the three periods was 30%, 22%, and 16% (*p* < 0.00001) (Figure 3A). In a univariate analysis, there was no significant effect of the year of transplant on TRM for patients with GvHD grade II (28%, 26%, and 23%, respectively, *p* = 0.3). In a multivariate analysis on TRM patients with GvHD grade II, positive predictive factors were transplants in 2000–2010 (RR 0.62, *p* < 0.0001) and 2011–2020 (RR 0.35 *p* < 0.0001), patients aged ≤50 years (*p* < 0.0001), and HLA identical sibling donors (Table 3). When selecting GvHD grades III–IV, TRM was 79%, 49%, and 58% in the three periods, with no change beyond the year 2010. In multivariate analysis, there was a significant reduction of TRM for the two more recent transplant periods as compared to transplants before the year 2000: 2000–2010 (RR 0.41, *p* < 0.00001) and year 2011–2020 (RR 0.45, *p* = 0.005).

**Table 3:** Multivariate Cox analysis on the risk of TRM in patients with GvHD grade II.

Variable	Baseline	Compared	RR	(95% CI)	*p*-value
Year of TX	<2000	2001–2010	0.62	0.39–0.78	<0.00001
		>2010	0.35	0.23–0.59	<0.00001
Prophylaxis	CSA + MTX	+ATG	1.18	0.98–1.42	0.07
		PTCY +CSA + MMF	0.88	0.40–1.90	0.7
Donor type	HLA id SIB	Fam.mismatched	1.68	1.01–1.67	<0.00001
		UD matched	1.45	1.69–2.61	0.001
		UD mismatched	1.8	1.24–2.60	0.0006
		UD cord blood	0.59	0.49–0.71	0.001
Donor age	≤40 years	>40 years	1.14	0.99–1.32	0.05
Diagnosis	SAA	Malignancies	1.61	1.18–2.20	0.002
Recipient age	≤50 years	>50 years	1.48	1.28–1.71	<0.00001

Year of Tx, year of transplant; CsA, cyclosporine; MTX, methotrexate; ATG, anti-thymocyte globulin; PTCY, post-transplant cyclophosphamide; UD, unrelated donor; CB, cord blood; SAA, severe aplastic anemia.

### 3.4 Overall survival

The actuarial 5-year survival for all patients was 47%, 51%, and 58% in the three time periods, respectively (*p* = <0.000001) (Figure 3B). In multivariate analysis on survival, predictive variables were a mismatched family donor (RR 1.65, *p* < 0.001), a matched unrelated donor (RR 1.36, *p* = 0.0007), a mismatched unrelated donor (RR 1.71, *p* < 0.00001), an unrelated cord (RR1.53, *p* = 0.0005), an advanced disease phase (RR 2.0, *p* < 0.00001), recipient age > 40 (RR1.28 *p* < 0.0001), the use of PTCY (RR 0.6, *p* = 0.003), year of transplant 2000–2010 (RR 0.80, *p* = 0.008), and year of transplant 2011–2020 (RR 0.50, *p* < 0.00001).

### 3.5 Overall survival in patients with GvHD grades II–IV

In univariate analysis, there was no effect of the year of transplant on survival for patients with GvHD grade II (52%, 44%, 56%, *p* = 0.3), and clinically non-significant change in survival for GvHD grade III–IV patients (15%, 30%, and 20%, *p* = 0.003), the most recent period being worse than the previous.

In a multivariate analysis on survival for patients with GvHD grade II, prognostic factors were disease phase beyond complete remission (RR 2.0, *p* < 0.00001) followed by patients age > 40 years (RR 1.53, *p* = 0.001), year of transplant 2011–2020 as compared to <2000 (RR 0.59, *p* = 0.01), and a family mismatched donor (RR1.50, *p* = 0.01). For patients with GvHD grades III–IV, predictive factors in multivariate analysis were the year of transplant 2000–2010 (RR 0.49, *p* = 0.0001), use of PTCY (RR 0.26, *p* = 0.002), patients age > 40 (RR 1.60, *p* = 0.008), a family mismatched donor (RR 1.83, *p* = 0.005), and donor age > 40 (RR 1.61, *p* = 0.01).

### 3.6 Relapse

The CI of relapse in the three periods for acute leukemia in the first complete remission was 23%, 29%, and 21% (*p* = 0.2). In a multivariate Cox analysis, GvHD had no protective effect on acute leukemia in first remission (RR 0.9, *p* = 0.7). Grades II–IV did have a protective effect on relapse in patients other than acute leukemia (RR 0.80, *p* = 0.003).

### 3.7 Causes of death

In patients with GvHD grade 0-I or II-IV the causes of death were the following: acute GvHD 1% and 17% respectively; infections 9% and 19%; chronic GvHD 1% and 9%; relapse 21% and 20% respectively.

## 4 Discussion

In 2010, the Seattle group reported a significant reduction in TRM, comparing two separate time periods (1993–1997 and 2003–2007): this was thought to be due, among others, to better GvHD prophylaxis and better management of infections (13). We have confirmed in this study that the cumulative incidence of acute GvHD has decreased with time, most possibly as a consequence of changes in GvHD prophylaxis. Survival of patients with acute GvHD grade II and grades III–IV has improved over time. Relapse has remained unchanged, and TRM has been reduced. As a consequence, there has been a significant improvement in overall 5-year survival with time.

As to the first finding, we were pleased to see a very significant reduction of acute GvHD, in both grades II–IV, and grades III–IV, despite older patients’ age, and greater use of alternative donor grafts: the reduction was very significant in the period 2001–2010 (as compared to <2000) (*p* < 0.00001) and also in the period 2011–2020 (*p* < 0.00001). In addition, we could document a significant delay in the median time to onset of GvHD from day 13 before the year 2000 to day 33 beyond the year 2010. We believe that the reduction of GvHD has occurred with changes in GvHD prophylaxis. The addition of ATG started in the late 90s, mostly for patients with unrelated donor transplants (14–16): in multivariate analysis, the addition of ATG to CSA MTX, results in a relative risk of grades II–IV GvHD of 0.65 (*p* < 0.00001) and a RR of 0.45 (*p* < 0.00001) for GvHD grades III–IV. The other major change in prophylaxis has been the addition of PTCY (11, 12, 16) but only for patients receiving a haploidentical transplant: It is rather surprising that, despite mismatched family grafts have a higher risk of GvHD grades II–IV and III–IV, PTCY shows a borderline significant protective effect of 0.71 (*p* = 0.06). Other variables predictive of GvHD are older donor age and matched unrelated grafts as compared to identical siblings. So the first conclusion is that GvHD prophylaxis has improved and we are currently running a 16% incidence of GvHD grades II–IV in our most recent period with 959 patients, and a 4% of GvHD grades III–IV. This is somewhat lower than what is seen in other studies: in a recent registry study on over 100.000 patients, the lowest rate of GvHD grades II–IV and III–IV is 28% and 11%, respectively, in the most recent period 2011–2015 (2).

If better prophylaxis has resulted in lower GvHD incidence, the question then is, has treatment also improved? In univariate analysis, looking at the impact of transplant era on TRM in patients with GvHD grade II, there was no significant change in TRM in the three periods (28%, 26%, and 23%, respectively, *p* = 0.3); similarly for patients with GvHD grades III–IV TRM was 76%, 48%, and 59%, respectively, with a reduction beyond the year 2000, but not further beyond the year 2010. However, we reasoned that patients’ age has significantly increased together with the use of alternative donors: both these two variables are negative predictors of TRM and survival. Thus, when looking at patients with grade II GvHD in a multivariate analysis, including patients’ age and donor type as covariates, the year of the transplant was a highly significant predictor of TRM, with a relative risk of 0.62 and 0.35 for the year 2000–2010 and 2011–2020, compared to patients grafted before year 2000. What has been the reason for reduced TRM, in patients with active GvHD, is difficult to assess: ruxolitinib (17) for the treatment of GvHD was not used currently before 2020, and clearly not before 2010. We have been using an anti-CD26 antibody in a significant number of refractory GvHD patients, with encouraging response rates (18). However, there has also been improvement in supportive care, and in anti-infectious therapy, which may have contributed to improved outcomes. In patients with GvHD grades III–IV, there was a significant reduction of TRM beyond the year 2000, but not really beyond the year 2010. Other predictive variables of TRM in patients with grades II and III–IV GvHD are younger patients’ age and an HLA identical sibling donor.

GvHD grades II–IV GvHD did not have a protective effect on relapse in patients with acute leukemia in first remission, nor acute leukemia with advanced disease. There was a protective effect of GvHD on relapse in patients other than acute leukemia (RR 0.81), but the detrimental effect on TRM was stronger (RR 1.43).

The third finding is reduced overall TRM, which paralleled reduced GvHD, despite increasing patients’ age and increasing use of alternative donors: We believe this has been the result of improved GvHD prophylaxis, together with improved supportive care, including diagnosis and treatment of bacterial, fungal, and viral infections. The lack of a protective effect of reduced intensity regimens may be due to the fact that older age is a negative predictor, and older patients are more likely to receive reduced intensity regimens. Overall the cumulative incidence of relapse has remained unchanged over time, and therefore, with a reduction of TRM, the 5-year survival has improved from 47% before year 2000, to 58% in the period 2011–2020. This figure is in keeping, with a report showing an overall 3-year survival of 54% (2). The limitations of this study include the lack of data on different organ involvement in patients with different severity of GvHD and the fact that no analysis was performed on chronic GvHD. We are planning a study on chronic GvHD, since some reports actually report an increase of this complication in recent years, especially due to the widespread use of peripheral blood as a stem cell source.

In conclusion, we confirm a reduced incidence and delayed onset of GvHD with time. Treatment has also improved as suggested by reduced TRM and improved survival, in a multivariate analysis, taking into consideration the current older patient population grafted from alternative donors. This is true for patients with GvHD grade II and grades III–IV. Nevertheless, a TRM in excess of 20% for GvHD grade II and in excess of 50% for GvHD grades III–IV, in the most recent transplant period, shows the need for more effective therapy. We shall see whether the use of new approved therapeutic options, such as ruxolitinib (17), will finally reduce transplant mortality in patients who develop GvHD.

## Data availability statement

The raw data supporting the conclusions of this article will be made available by the authors, without undue reservation.

## Ethics statement

Ethical approval was not required for the study involving humans in accordance with the local legislation and institutional requirements. Written informed consent to participate in this study was not required from the participants or the participants’ legal guardians/next of kin in accordance with the national legislation and the institutional requirements. The patients signed an informed consent to share data of their transplant with local, National and International organizations, according to the requirements of the National Regulatory Agency.

## Author contributions

ADi: Writing – original draft. AMR: Writing – review & editing. ADo: Writing – review & editing. CD: Writing – review & editing. FG: Writing – review & editing. MTV: Writing – review & editing. SB: Writing – review & editing. PC: Writing – review & editing. LL: Writing – review & editing. FS: Writing – review & editing. SG: Writing – review & editing. EM: Writing – review & editing. AF: Writing – review & editing. SS: Writing – review & editing. EA: Writing – review & editing. AB: Writing – original draft.

## References

[1] Malard F Holler E Sandmeier B Huang H Mohty M. Acute graft versus host disease. Nat Rev. (2023) 27:1–18. doi: 10.1038/s41572-023-00438-1 37291149

[2] Greinix HT Eikema DJ Koster L Penack O Yakoub-Agha I Montoto S. Improved outcome of patients with graft-versus-host disease after allogeneic hematopoietic cell transplantation for hematologic malignancies over time: an EBMT mega-file study. Haematologica. (2022) 107:1054–63. doi: 10.3324/haematol.2020.265769, PMID: 34162176 PMC9052930

[3] Nassereddine S Rafei H Elbahesh E Tabbara I. Acute graft versus host disease: a comprehensive review. Anticancer Res. (2017) 37:1547–55. doi: 10.21873/anticanres.11483 28373413

[4] Ferrara JLM Chaudhry MS. GVHD: biology matters. Blood Adv. (2018) 2:3411–7. doi: 10.1182/bloodadvances.2018020214, PMID: 30482771 PMC6258915

[5] Ruggeri A Labopin M Bacigalupo A Afanasyev B Cornelissen JJ Elmaagacli A. Post-transplant cyclophosphamide for graft-versus-host disease prophylaxis in HLA matched sibling or matched unrelated donor transplant for patients with acute leukemia, on behalf of ALWP-EBMT. J Hematol Oncol. (2018) 11:40–9. doi: 10.1186/s13045-018-0586-4, PMID: 29544522 PMC5855999

[6] Filipovich AH Weisdorf D Pavletic S Socie G Wingard JR Lee SJ. National institutes of health consensus development project on criteria for clinical trials in chronic graft-versus-host disease: I. Diagnosis and staging working group report. Biol Blood Marrow Transplant. (2005) 11:945–56. doi: 10.1016/j.bbmt.2005.09.004, PMID: 16338616

[7] Saliba RM de Lima M Giralt S Andersson B Khouri IF Hosing C. Hyperacute GVHD: risk factors, outcomes, and clinical implications. Blood. (2007) 109:2751–8. doi: 10.1182/blood-2006-07-034348, PMID: 17138825

[8] Glucksberg H Storb R Fefer A Buckner CD Neiman PE Clift RA. Clinical manifestations of graft-versus-host disease in human recipients of marrow from HL-A-matched sibling donors. Transplantation. (1974) 18:295–304. doi: 10.1097/00007890-197410000-00001, PMID: 4153799

[9] Harris AC Young R Devine S Hogan WJ Ayuk F Bunworasate U. International, multicenter standardization of acute graft-versus-host disease clinical data collection: a report from the Mount Sinai Acute GVHD International Consortium. Biol Blood Marrow Transplant. (2016) 22:4–10. doi: 10.1016/j.bbmt.2015.09.001, PMID: 26386318 PMC4706482

[10] Ruutu T Gratwohl A de Witte T Afanasyev B Apperley J Bacigalupo A. Prophylaxis and treatment of GVHD: EBMT–ELN working group recommendations for a standardized practice. Bone Marrow Transplant. (2014) 49:168–73. doi: 10.1038/bmt.2013.107, PMID: 23892326

[11] Luznik L O’Donnell PV. Simons HJ et al HLA-Haploidentical bone marrow ransplantation for hematological malignancies using nonmyeloablative conditioning regimen and high dose, posttransplantation cyclophosphamide. Biol Blood Marrow Transplant. (2008) 14:641–50. doi: 10.1016/j.bbmt.2008.03.005, PMID: 18489989 PMC2633246

[12] Bolaños-Meade J Hamadani M Wu J Al Malki MM Martens MJ Runaas L. BMT CTN 1703 investigators. post-transplantation cyclophosphamide-based graft-versus-host disease prophylaxis. N Engl J Med. (2023) 388:2338–48. doi: 10.1056/NEJMoa2215943 37342922 PMC10575613

[13] Gooley TA Chien JW Pergam SA Hingorani S Sorror ML Boeckh M. Reduced mortality after allogeneic hematopoietic-cell transplantation. N Engl J Med. (2010) 363:2091–101. doi: 10.1056/NEJMoa1004383, PMID: 21105791 PMC3017343

[14] Finke J Bethge WA Schmoor C Ottinger H Stelljes M Volin L. Standard graft-versus-host disease prophylaxis with or without anti-T-cell globulin in haematopoietic cell transplantation from matched unrelated donors: a randomised, open-label, multicentre phase 3 trial. Lancet Oncol. (2009) 10:855–64. doi: 10.1016/S1470-2045(09)70225-6 19695955

[15] Walker I Panzarella T Couban S Couture F Devins G Elemary M. Pretreatment with anti-thymocyte globulin versus no anti-thymocyte globulin in patients with haematological malignancies undergoing haemopoietic cell transplantation from unrelated donors: a randomised, controlled, open-label, phase 3, multicentre trial. Lancet Oncol. (2016) 17:164–73. doi: 10.1016/S1470-2045(15)00462-3, PMID: 26723083

[16] Bacigalupo A Lamparelli T Bruzzi P Guidi S Alessandrino PE di Bartolomeo P. Antithymocyte globulin for graft-versus-host disease prophylaxis in transplants from unrelated donors: 2 randomized studies from Gruppo Italiano Trapianti Midollo Osseo (GITMO). Blood. (2001) 98:2942–7. doi: 10.1182/blood.V98.10.2942, PMID: 11698275

[17] Zeiser R von Bubnoff N Butler J Mohty M Niederwieser D Or R. Ruxolitinib for glucocorticoid-refractory acute graft-versus-host disease. New Engl J Med. (2020) 382:1800–10. doi: 10.1056/NEJMoa1917635, PMID: 32320566

[18] Bacigalupo A Angelucci E Raiola AM Varaldo R Di Grazia C Gualandi F. Treatment of steroid resistant acute graft versus host disease with an anti-cd26 monoclonal antibody- begelomab. Bone Marrow Transpl. (2020) 55:1580–7. doi: 10.1038/s41409-020-0855-z, PMID: 32203257

